# The Orthodox Dry Seeds Are Alive: A Clear Example of Desiccation Tolerance

**DOI:** 10.3390/plants11010020

**Published:** 2021-12-22

**Authors:** Angel J. Matilla

**Affiliations:** Departamento de Biología Funcional (Área Fisiología Vegetal), Facultad de Farmacia, Universidad de Santiago de Compostela, 15782 Santiago de Compostela, Spain; angeljesus.matilla@usc.es; Tel.: +34-981-563-100

**Keywords:** orthodox seeds, *Medicago truncatula*, desiccation tolerance, resurrection plants, *Xerofita viscosa*, cell wall, *ABI3*, *Physcomitrella patens*, LEA proteins, sHSPs, glass state, *Craterostigma plantagineum*

## Abstract

To survive in the dry state, orthodox seeds acquire desiccation tolerance. As maturation progresses, the seeds gradually acquire longevity, which is the total timespan during which the dry seeds remain viable. The desiccation-tolerance mechanism(s) allow seeds to remain dry without losing their ability to germinate. This adaptive trait has played a key role in the evolution of land plants. Understanding the mechanisms for seed survival after desiccation is one of the central goals still unsolved. That is, the cellular protection during dry state and cell repair during rewatering involves a not entirely known molecular network(s). Although desiccation tolerance is retained in seeds of higher plants, resurrection plants belonging to different plant lineages keep the ability to survive desiccation in vegetative tissue. Abscisic acid (ABA) is involved in desiccation tolerance through tight control of the synthesis of unstructured late embryogenesis abundant (LEA) proteins, heat shock thermostable proteins (sHSPs), and non-reducing oligosaccharides. During seed maturation, the progressive loss of water induces the formation of a so-called cellular “glass state”. This glassy matrix consists of soluble sugars, which immobilize macromolecules offering protection to membranes and proteins. In this way, the secondary structure of proteins in dry viable seeds is very stable and remains preserved. ABA insensitive-3 (ABI3), highly conserved from bryophytes to Angiosperms, is essential for seed maturation and is the only transcription factor (TF) required for the acquisition of desiccation tolerance and its re-induction in germinated seeds. It is noteworthy that chlorophyll breakdown during the last step of seed maturation is controlled by ABI3. This update contains some current results directly related to the physiological, genetic, and molecular mechanisms involved in survival to desiccation in orthodox seeds. In other words, the mechanisms that facilitate that an orthodox dry seed is a living entity.

## 1. Dormancy and Drying: Two Key Traits along Seed Evolution

The colonization of, and permanence in, land by plants was one of the most important events in the history of our planet [1]. The evolution of this event included significant transformations such as the occurrence of a diploid sporophyte, transition from gametophyte-dominant to sporophyte-dominant, and development of many specialized tissues and organs (i.e., flowers, leaves, roots, seeds, stomata, and vascular tissues) [2]. The evolutionary success of higher plants consists of their ability to produce seeds, propagules responsible for reproduction, dispersal, and survival. The seeds have undergone outstanding morphological and physiological changes through evolution [3,4,5,6]. Thus, we can refer to the storage tissues to support the embryo growth, and the existence of protective layers of hardened tissues to prevent desiccation [6,7,8,9,10]. After fertilization, the three main parts of the seed, embryo, endosperm, and seed coat undergo a series of developmental processes that converge in the production of a mature seed that is developmentally arrested, strongly desiccated, and metabolically quiescent [11,12]. This dormancy state constitutes an adaptive trait limiting germination under environmental conditions that would promote optimal germination in non-dormant seeds. Seed dormancy and the subsequent germination are controlled by internal cues and environmental signals [12,13,14].

Dormancy is a strategy that allows certain organisms to survive through conditions that are suboptimal for growth and reproduction by entering a reversible state of reduced metabolic activity [11,12]. Plants use the seed dormancy to move through time and space. Therefore, the acquisition of dormancy during seed maturation has been a considerable evolutionary advance [15,16,17,18]. The degree and kind of dormancy is under strong natural selection. On the other hand, dormancy is an outstanding agronomic trait related to seed quality and grain yield [19]. Conifers may have been the oldest plants to show this strategy in addition to showing greater physiological tolerance to water stress [15]. Currently, the ancestral seed dormant state is not known with certainty. However, the morphophysiological dormancy, in conflict with physiological dormancy, can be a solid candidate [17,20]. It is also unclear whether all dormancy classes can emanate from each other or, alternatively, whether certain classes necessarily precede others. There is growing evidence that at least some of the molecular steps that control dormancy (e.g., *DOG1*) are common among seed plants [21,22,23,24]. Currently, although there are several clues in some organs other than seeds [2,25], the evolution and genomic consequences of seed dormancy is not yet known with certainty. Two questions can be representatives of this gap: does natural selection act on seed dormancy genes and what are these genes; and what are the interactions between seed dormancy and other evolutionary forces? [12,17,26].

To rationally progress in the study of seed dormancy it is paramount to identify the QTLs related and thus dissect the molecular basis of this trait. At present, many QTLs affecting seed dormancy have been detected in model and crops plants such as Arabidopsis [24], barley [27], and sorghum [28]. It must be kept in mind that the seed goes into a dormant state once a significant loss of water occurs (i.e., desiccation). Anhydrobiotic organisms as seeds achieve dormancy by replacing most of the cell water by compatible osmolytes, thereby transforming the cytoplasm into a metastable “glass state”. However, the loss of water does not have the same intensity in all tissues of seeds (e.g., embryo). This delicate organ is protected from drying out by special mechanisms. These mechanisms, some of which are summarized in this review, involve the late embryogenesis abundant (LEA) and heat-shock protein (*sHSP*) genes, and ABA signaling pathway genes (e.g., *ABI3*) [29,30,31,32,33,34]. ABA was shown to activate the synthesis of LEA proteins, which impact on desiccation tolerance of developing seeds and seed tolerance to adverse environment factors [34,35].

This review contains current views directly related to physiological and molecular mechanisms involved in survival of severe desiccation in seeds. In other words, this review attempts to clarify the mechanisms of orthodox seeds that explain how life can continue in extreme conditions, including lack of water.

## 2. Seed Desiccation Tolerance: A Trait to Colonize Terrestrial Ecosystems

Water is essential for all living organisms since it provides structural order to cells, stabilizes macromolecules, and maintains a cellular microenvironment within which critical metabolic systems remain and chemical reactions occur [36,37]. Accordingly, water loss leads to cell plasmolysis and increases viscosity of cytoplasm thus generating mechanical stress provoked by cell shrinkage [38]. To prevent this cellular stress and stay alive, evolution allowed plants to adapt to suitable hydrated environments. Desiccation tolerance is the ability to dry to equilibrium with moderated dry air (50% or lower relative humidity) and resume metabolism when rehydrated [39]. This feature is shared by taxa not phylogenetically related to plants (e.g., tardigrades, fungi, mosses, and ferns), and is also found throughout the whole life cycle of an organism or parts of the same (e.g., flowering plants, many of which produce desiccation-tolerant seeds, whereas their sporophytes are only very rarely desiccation tolerant) [40]. The acquisition of desiccation tolerance was essential for the colonization of terrestrial habitats by the primitive plants and the routes that control this trait are likely ancestral and conserved in most Angiosperms. Nevertheless, the damage caused by extreme hydration fluctuations must be at a retrievable level [41]. In summary, the ability to dry without dying is a fundamental characteristic, which enabled early terrestrial plants to colonize terrestrial ecosystems [34].

Desiccation-tolerant cells can avoid or resist harmful effects derived from mechanical stress through increased vacuolation and/or cell wall (CW) folding [42]. Most plants, including crops (i.e., drought-avoiding species), can rarely survive water potentials of less than −4 MPa. Although the seeds of higher plants withstand desiccation (water content of 8–10%), vegetative tissues of the majority do not tolerate water content below 30–60% [43]. Vegetative desiccation tolerance presumably evolved from pre-existing seed desiccation [44]. Unlike bryophytes and liquids, higher plants rarely have desiccation tolerance in their vegetative tissues. Probably, the ancestral desiccation-tolerance mechanisms in vegetative tissues were chosen by early tracheophytes for abiotic stress response and protection of their reproductive organs. Desiccation-tolerance was originally present in chlorophytes algae that were forerunners of the basal land plants (i.e., bryophytes) [45]. On the other hand, drying cells to 50% relative humidity leads to metabolic arrest since cellular water content is below the level required to form an aqueous monolayer around macromolecules [46]. Desiccation-tolerant vegetative tissues are completely absent in Gymnosperms (e.g., conifers). In many Angiosperms, vegetative-desiccation tolerance has been lost. However, most of the species that maintained this tolerance in their seeds are considered orthodox [47]. Over 95% of Angiosperms produce orthodox seeds [4]. By contrast, the recalcitrant ones are desiccation sensitive and released from the mother plant under conditions immediately conducive to triggering germination. How the differences between fully orthodox and fully recalcitrant seeds are generated to respond to dryness is still an unknown that needs to be clarified [48]. Evolutionarily, the classification of seeds as fully orthodox and recalcitrant must be taken with care, given that the existence of intermediate seeds must be considered. That is, a gradient between the most tolerant seeds and the most sensitive ones must be taken into account when discussing results.

## 3. Desiccation Tolerance in Resurrection Plants

### 3.1. Background

Although the core of desiccation-tolerance responses is retained in orthodox seeds, this special adaptation is only present in the vegetative organs of non-vascular plants (i.e., bryophytes) and few Angiosperm species (<0.15%; about 300 species) commonly known as resurrection plants [32,47,48,49]. That is, seeds and vegetative organs of resurrection plants are tolerant to desiccation and survive under water potentials of −100 MPa (i.e., 1–5% relative water content) and even lower [43,50]. Among other essential functions, resurrection plants resume photosynthetic activity and growth within a few hours after re-watering. Thus, a resurrection plant tolerates dehydration without affecting their vital attitudes. Phylogenetic evidence suggests that resurrection plants regained the ability to tolerate desiccation in their vegetative tissues through mechanisms present first in bryophytes [51]. Therefore, it is probable that almost all resurrection plants have reactivated in their vegetative parts the genetic specific program of orthodox seed desiccation tolerance [52]. In other words, presumably, the evolution of vegetative desiccation tolerance in resurrection plants occurred through co-option of the desiccation-tolerance mechanisms already active in seeds [32].

The regulatory interactions activating desiccation tolerance remain largely unknown. In order to control seed desiccation tolerance, the transcription factors (TFs) *PLATZ1* and *PLATZ2* (i.e., plant-specific zinc-dependent transcription repressors) and *AGL67* (i.e., AGAMOUS- LIKE67) act downstream of the embryo development regulatory master genes LAFL and are essential for the acquisition of desiccation tolerance in Arabidopsis seeds [53,54]. However, are these three genes conserved in Angiosperms? It is widely known that *LEC1* and *ABI3* are highly conserved from bryophytes to Angiosperms [54]. Interestingly, mutations in *LEC1*, *ABI3*, and *FUS3* drastically affect desiccation tolerance. On the other hand, the vegetative desiccation tolerance in the resurrection plant *Xerophyta humilis* has not evolved through reactivation of the seed canonical LAFL network [41,55]. Transcriptome studies showed that the mechanisms involved in desiccation tolerance are conserved in resurrection plants, seeds, and pollen [56].

Together, investigating the mechanisms of desiccation tolerance in resurrection plants leads to understanding possible pathways for the evolution of desiccation tolerance in plants. To progress in this knowledge, the genomes of several species were recently sequenced and used as a model to understand the numerous interacting factors promoting desiccation tolerance. These studies include: (i) the lycophytes *Selaginella lepidophylla* [57] and *S. tamariscina* [58]; (ii) the monocots grass *Oropetium thomaeum* [59], and poikilochlorophyllous *Xerophyta viscosa* which destroyed the photosynthetic apparatus during dehydration and recovers it upon re-watering [44,52]; and (iii) the extremophile dicot *Boea hygrometrica* [31]. Genetic studies in *X. viscosa* are noteworthy since they have supplied valuable information on the genetic basis of desiccation tolerance [52]. These genomic advances provided profitable resources into the evolution of vascular plants and how resurrection plants acquired desiccation tolerance. In short, the molecular physiology and genomic and transcriptomic data from these model plants facilitate a wealth of information to study the conserved mechanisms of desiccation tolerance [49,58,60]). In dry viable seeds, almost all mRNAs are preserved as ribonucleoproteins to make them immediately available upon rehydration. However, it is not yet documented if resurrection plants use similar post-transcriptional and translational control mechanisms in prevision of rehydration. A detailed review on the transcriptomics and metabolomics of resurrection plants was recently published [61].

### 3.2. Cell Wall Alterations

Desiccation tolerance is a convoluted multigenic and multifactorial process comprising a combination of structural and macromolecular changes, being the CW of the compartments involved. The CW is a highly dynamic cellular component [62], and its structure and composition are altered (i.e., remodeling) during cellular water loss [42]. Reactive xoygen species (ROS) are important molecules for the CW remodeling process given that they participate in compromised enzymatic reactions and work as signaling molecules [63,64]. Comparing the genomes of *Selaginella tamariscina* (desiccation tolerant) and *S. moellendorffii* (desiccation sensitive) demonstrated that the number of ROS-producing genes is much lower in the first lycophyte [58]. Interestingly, it was proposed that the ROS detoxification mechanisms within CW are less efficient than the intracellular ones because they rely on low levels of CW peroxidases [65]. In some resurrection plants, the peroxidase activities are highly increased upon re-watering but do not change during dehydration [66]. In *X. viscosa*, peroxidases are up-regulated during dehydration, but down-regulated upon rehydration, which is a prerequisite for CW stiffness under drought [67]. In other words, low peroxidase activities tend to generate hydroxyl radicals which lead to CW loosening. On the contrary, high amounts of peroxidases facilitate CW stiffness [68,69]. In parallel with these studies, screenings for foliar proteins interacting with dehydration induced CW proteins in resurrection of *C. plantagineum* identified a germin-like protein (CpGLP1). This CpGLP1 accumulates in the CW of desiccating leaves, binds with pectins, and has superoxide dismutase (SOD) activity [70]. The authors claim a role for this enzymatic activity in the ROS metabolism related to the control of CW plasticity during desiccation. It is noteworthy that the *OsGLP2-1* expression is increased in response to ABA and involved in the regulation of rice seed dormancy [71]. Interestingly, the balance between the ABA and GA signals during seed germination is regulated by functional proteins such as *OsGLP2-1*, which bind to the promoters of *ABI5* and *GAMYB* [71].

Cell shrinkage, as water is lost, has negative effects on cellular structure and function. To adapt to this contraction stress, both the CW and plasmalemma fold, enabling maintenance of the membrane and the CW surface area, which is critical for cells to survive rehydration without breaking [72,73]. CW folding is a tightly regulated reorganization of the CW structure resulting in increased flexibility, allowing the CW to adjust to the reduced volume of desiccated cells more easily [74]. During the dehydration of *Craterostigma wilmsii*, a strong folding of CW takes place in foliar tissues, and a decrease of about 78% of the cell volume occurs. The CW folding is considered as an ability to maintain the contacts between the plasmalemma and the CW during dehydration and avoid the tearing between these structures, and hence cell lysis and death (see Table 2 from [49]). In later development, this stressful mechanical effect is minimized in both orthodox seeds and resurrection plants by CW folding and accumulation of dry matter to replace lost water. Therefore, unstructured LEA proteins, sugars, storage proteins, and simple polypeptides are accumulated [75]. These accumulated molecules likely also play a role in simple mechanical stabilization of desiccating cells [34]. In orthodox seeds, the large central vacuole fragments into multiple and more mechanically stable vacuoles, generally filled with storage proteins or compatible solutes. This vesiculation, which avoids the vacuole rupture, also occurs in resurrection plants. The vacuolar content can range from protein to metabolites and is heavily species dependent [44]. The folding process similarly occurs in both embryos and desiccating resurrection plants [76]. However, it is not clear whether the folding process and its regulation are similar in embryos and the leaves of resurrection plants subjected to dehydration. In resurrection plants, CW extensively shrinks and folds upon desiccation, but the integrity and continuity of CW structures are maintained and restored when tissues are re-watered [60,76].

The CW structural components were recently addressed in leaves of some resurrection plants [49,77]. Although the protective function of cellulose during water stress has been well studied, there is no information available about the relationship between cellulose and desiccation. However, high levels of de-methyl esterified homogalacturonan were found upon desiccation and the levels being reversed after re-watering. In *C. plantagineum* and *C. wilmsii*, xyloglucan and xylan, two cellulose-linking CW components, increased upon desiccation [77,78]. Likewise, in all studied resurrection plants, the changes in the pectin composition led to a more rigid CW upon dehydration [42]. De-methyl esterified pectin increases in the CW of *C. wilmsii*, *C. plantagineum*, and *L. brevidens*, which is probably due to pectin methyl esterase activities during dehydration [77]. In summary, desiccation-tolerant plants have an extraordinary ability to regrow when re-irrigated because they have an aptitude to alter the leaf structure, and modify both proteins and cellulosic and non-cellulosic polymers of CW. Finally, Table 1 from [42] summarizes the dehydration-induced changes in the expression of genes encoding proteins and enzymes that modify the CW in resurrection species. On the other hand, given that cellular turgency mechanically affects to CW, mechanosensors located in this structure detect alterations in the cell turgor [79]. Some of these sensors involved in dehydration were recently studied [80].

The transcriptome analysis in *C. plantagineum* revealed elevated expression of genes encoding pore Ca^++^ channels and others undefined upon dehydration (Figure 2 from [42]). Multi-omic analysis showed that several CW-related genes involved in processes such as the regulation of CW plasticity, organization, and dynamics are differentially modulated upon dehydration. These analyses suggest the importance of CW remodeling during the acquisition of desiccation tolerance [40,81,82]. In conclusion, the activation in resurrection plants of metabolic routes leading to the increase in the flexibility of the CW structural components, as well as the increase in CW stability, suggests a tightly controlled folding process during dehydration that finally keeps the plasmalemma and photosynthetic apparatus intact. The study of these aspects in the seed will provide new reasons for understanding the process of tolerance to desiccation, a puzzle of enormous complexity.

## 4. Acquisition of Desiccation Tolerance: An Essential Trait in Orthodox Seeds

As indicated before, desiccation tolerance appeared very early in the evolution of terrestrial life and was likely present in the ancestor of all land plants [83]. During maturation of orthodox seeds, physiological dormancy is triggered to avoid viviparism (i.e., preharvest sprouting). Seed desiccation tolerance is acquired in parallel to reserve accumulation gathering of metabolites before desiccation tolerance takes place, and finally, the dry and viable seeds with a determined level of longevity are formed [13,84,85,86]. Thus, oleosins, caleosins, and seed storage proteins act to stabilize oil bodies and fill cell volume, increasing the tolerance of cells to desiccation and serving to protect/act as energy reserves for germination [34,87]. Likewise, during maturation considerable changes occur at omic levels [85,88]. On the other hand, an excellent review by Oliver et al. (2020) provides current data on the preservation of the photosynthetic apparatus during desiccation [41]. At an evolutionary level, desiccation and longevity provide in orthodox seeds a highly effective strategy for successful transmission of genetic information from the mother plant to the next generation. However, the cause-and-effect relationship between seed longevity and chlorophyll content remains elusive [4,89,90].

The desiccation tolerance in orthodox seeds is acquired as a pre-programmed developmental process rather than as a physiological response to water deficit [34]. Usually, the seed irreversibly loses their desiccation tolerance in the transition from phase II to III (see Figure 1 from [44,91]). Maternal ABA, accumulation of reserves, heat shock, and late embryogenesis abundant proteins (sHSPs and LEAs, respectively) are involved in the acquisition of seed desiccation tolerance and induction of primary dormancy [92,93,94,95]. The orthodox seeds can survive long time periods in a desiccated, stored, and quiescent state, allowing time for dispersal [12,48,73,96]. As a representative example, the germination of palm (*Phoenix dactylifera*) orthodox seeds, with highlighted antiqueness (i.e., 2000 years old, radiocarbon age) that originated in an environment with high temperatures and dry soil, confirmed that these ancient reproductive propagules already had the molecular tools necessary to survive and maintain their viability in a desiccated state [97]. Accordingly, in addition to being desiccation tolerant, these ancient seeds also acquired remarkable longevity [98]. Studies on lotus (*Nelumbo nucifera*) revealed striking seed viability of more than 1300 years, which was attributed to the presence of several thermostable proteins, including sHSPs [99]. At present, it is known that throughout long-term storage, the seed longevity depends on moisture content, relative humidity, O_2_ pressure, and temperature [100]. Longevity is gradually acquired from seed filling onwards [85,101,102]. However, the mechanism by which anhydrobiotic longevity is improved is still unknown. On the other hand, during dry storage, seed viability gradually decreases due to aging processes and/or deterioration events.

Seed desiccation tolerance is the ability to resist water loss (i.e., more than 90% of the total water content) by suspending growth and development resuming normal metabolism after rehydration without accumulating lethal damage in tissues [44,98,103]. This ability is found in all groups of living organisms and includes microorganisms and a few species of animals [104,105,106,107]. Seed desiccation tolerance is an adaptive strategy to enable seed survival during storage or environmental stress and ensures better dissemination of the species in terrestrial habitats [108,109,110,111]. Therefore, in anhydrobiote organisms (i.e., organisms that remain in the absence of water) the cytoplasm does not have free water—a requirement whereby most of the cellular water is bound to macromolecules [112,113]. Perhaps resurrection plants and fully orthodox seeds employ similar mechanisms to deal with extreme water loss [44]. In any case, resurrection plants and orthodox seeds serve as a paramount model to understand the interacting factors promoting desiccation tolerance [60]. Interestingly, desiccation tolerance can be re-induced in germinated orthodox seeds in the presence of ABA. Therefore, this peculiarity is successfully used to understand the mechanisms linked to desiccation tolerance in developing seeds [114,115,116,117,118,119,120,121].

During drying and re-watering of seeds, a complex array of structural, metabolic, chemical, mechanical, and molecular changes take place to prevent lethal cellular damage. Thus, to be desiccation tolerant a seed must: (i) limit its damage during desiccation; (ii) maintain physiological integrity in the dry state; and (iii) repair damage upon rehydration to regain the integrity of membranes, membrane-bound organelles, and genetic material [122]. To prepare for dry state, developing seeds need to generate protective mechanisms to provide desiccation tolerance and life span. The question of how dry seeds can remain viable for a long time lies in resolving how the integrity of DNA is protected. Some types of DNA damage are carried out in aqueous media. Therefore, the water interacting with the DNA needs to be removed or decreased to prevent damage. Some oligosaccharides (i.e., raffinose family) act as DNA protective agents against hydrolytic damage via a water replacement mechanism [34,53,110]. Other mechanisms comprise a set of the genes encoding protective molecules such as enzymes involved in scavenging ROS [123], osmotically active LEA proteins [124,125], sHSPs [30,126,127,128,129], various other stress proteins [130], and a set of antioxidant defenses against oxidative stress, such as glutathione (GSH) [131], tocopherols [132], and flavonoids present in the testa [6].

Although the relationship between LEA proteins and some oligosaccharides with desiccation tolerance seems to have been proven, the role of disordered sHSPs in seed desiccation tolerance and longevity has not been well elucidated. Multiple sHSP classes accumulate and are present in dry seeds but disappear rapidly upon germination. sHSPs are also hypothesized to play a role in the glassy matrix formation coincident with their presence in late embryo maturation [133,134,135,136]. As a mechanism of action, sHSPs are known to hold proteins and prevent their irreversible aggregation in addition to assisting in protein folding and helping with intracellular transport [137,138]. However, recently it was demonstrated that *OsHSP18.2*-mRNA is markedly increased at the late maturation stage, is highly abundant in dry seeds, and is probably involved in desiccation-tolerance of orthodox seeds [127]. High transcript accumulations of various sHSPs during seed maturation and in dry seeds were also reported in several plant species [127,139,140]. Taken together, the data obtained by the Sakar’s group suggest that OsHSP18.2 might protect vulnerable cellular proteins during maturation drying, desiccation, and aging in seeds by restricting ROS accumulation. It is important to point out that many of these protective molecules are absent in both non-seed tissues and recalcitrant seeds, suggesting that they have a unique function related to desiccation tolerance [48,141]. Finally, we should not rule out the role of DOG1 in maintaining a dry and viable seed in a live state during the desiccation-tolerance phase [24,142].

On the other hand, it remains unclear how many of the mechanisms that operate during the dehydration process can occur in seed tissues. One possible explanation could be a differential distribution of water between seed structures and individual biomolecules within them [143]. The referred protective mechanisms have previously been considered seed-specific, but it is now becoming clear that they were co-opted for vegetative desiccation tolerance (i.e., “genomic footprint” or clusters of desiccation-associated genes) [44,144]. In other words, we have been provided with strong evidence for the hypothesis that vegetative desiccation tolerance emerged by re-direction of genetic information from desiccation-tolerant seeds [44]. Thus, most current metabolic and transcriptional evidence is consistent with this hypothesis. However, the exact mechanisms by which seed-specific gene networks are induced in vegetative tissues remain a mystery.

## 5. The Protective Role of Intrinsically Disordered LEA Proteins

Desiccation-tolerant cells may contain up to 20% of intrinsically disordered proteins (i.e., LEAs and sHSPs). LEAs (hydrophylins) are a conserved group of highly hydrophilic and unstructured proteins widely distributed in the plant kingdom. In the hydrated state they are mainly in an unstructured conformation, whereas upon drying, many LEA proteins readily adopt a more structured conformation (i.e., acquiring α-helical structure). They basically arise during the seed maturation stage, but their mRNAs are detected 10–20 days earlier, suggesting a post-transcriptional regulation of abundance [85,88]. LEAs have a conservative three-dimensional structure and are mainly localized in the cytoplasm and nucleus to protect cellular structures that participate in the tolerance to water deficit thus keeping the molecules in hydration state. Evidence is accumulating that LEAs stabilize proteins to prevent stress-induced denaturation and aggregation of membrane and cytoplasmic proteins [145]. Interestingly, only some LEA proteins possess remarkable anti-aggregation properties [145]. In other words, additional protection in orthodox seeds is mediated by LEA proteins through the prevention of aggregation of cellular constituents as water is withdrawn and the distance between macromolecules diminishes [145,146]. The fact that LEA proteins have structural plasticity raise questions on whether water deficits modulate their conformation and if these possible changes are related to their function.

In the Arabidopsis genome, 51 genes encoding LEA proteins were identified [147], whereas in resurrection of *X. viscosa* there were 21 and two have been functionally characterized [60]. Dehydrins represent the most studied group of LEA proteins [148]. The decrease in dehydrins during seed germination can enhance sensitivity to desiccation [148,149]. The expression of *LEA* genes is much higher in seeds than in vegetative organs [150]. The accumulation of specific LEA proteins during seed development might be the key to obtaining desiccation tolerance [151,152]. Initially associated with seed desiccation, LEA proteins also promote desiccation tolerance in vegetative tissues of resurrection plants [153,154]. Although LEAs are present in orthodox dry seeds maintaining cellular structures, their accumulation decreases in germinating seeds when they become desiccation sensitive [151]. However, the role of specific LEA proteins during seed germination is not yet known. A detailed temporal proteome analysis during seed germination will help shed light on their importance. The occurrence of LEA proteins in recalcitrant seeds strongly suggests these proteins may be necessary but not sufficient for the acquisition of desiccation tolerance [29]. To undo this scientific uncertainty, it is very important to know intermediate species between orthodox and recalcitrant. Such a study, not yet conducted, will be key to understanding the aforementioned. Interestingly, silencing dehydrins in Arabidopsis does not affect desiccation tolerance, nor does the lack of one or two *LEA* genes, suggesting that not all LEA proteins are involved in the acquisition of seed desiccation tolerance [152].

LEA proteins have also been detected in seedlings, stems, leaves, and roots in response to abiotic stresses including drought, salinity, heat, and cold. *LEA* genes are also found outside the plant kingdom (e.g., anhydrobiotic bacteria and invertebrates), suggesting a common mechanism of desiccation tolerance across distinct life forms [155,156]. Due to its structure, LEA proteins are highly hydrated and during cell dehydration act as chaperons stabilizing denatured proteins promoting their refolding [151]. That is, LEA proteins form a water hydration shell around the molecules, a phenomenon made possible by their disordered structure. This structure aids water retention, preserving the three-dimensional structure of enzymes and other cellular components, thus preventing denaturation [157,158]. On the other hand, lipid bilayer membranes are also protected by LEA proteins; for example, pea mitochondrial LEA proteins interact with the negatively charged phosphate groups of dry membrane phospholipids to maintain their fluid crystalline state, thus increasing their stability [159]. This protective role is not general among all LEA proteins. Thus, certain homologs interact with membranes but do not increase its stability [160]. As will be indicated later, the interaction of LEA proteins with sugars produces a more stable cytoplasmic glass, providing further structural support during desiccation [161]. Upon further drying, some LEA proteins could participate in the glass formation in combination with sucrose and oligosaccharides [162]. EM6 specific LEA protein plays a key role in water binding during Arabidopsis seed maturation. The absence of EM6 may affect the stability of the glassy state of the cytoplasm, caused by increased water absorption, and thus may lead to the disintegration of membrane structures [163].

LEA proteins have also been implicated in the protection of chromatin structure during desiccation and are thought to play a role in DNA repair and chromatin remodeling [157]. In summary, the ability of LEA and other proteins to perform more than one function is included in “moonlighting” activity [157]. At an evolutionary level, the presence of genes for ancient LEA proteins in algal genomes suggests that the evolution of preexisting LEA families (including seed maturation proteins) and the formation of new LEA gene families has facilitated the colonization of terrestrial habitats [158,164]. Likewise, in comparing transcriptomes in desiccation-tolerant species during de- and rehydration a common set of genes and their orthologs associated with cellular protection and rehydration was observed [44,116]. Given that the production of LEA proteins has been positively and tightly correlated with the preparations for the entry of the seed into a desiccation-tolerant state, and decreases during germination, it would not be unreasonable to suppose that the cell has a mechanism to control the levels of LEA proteins. While the decrease in the LEA is well documented, there are few data on how such a specific drop occurs. It is unknown if LEA is required during onset imbibition, if the stored-mRNA participates in its synthesis, or if the transcription of its mRNA takes place at the beginning of imbibition [165,166,167,168]. These aspects are worth studying further. On the other hand, it is interesting to note that some *LEA* genes are under epigenetic control and transcription of them is rapidly reduced [164].

## 6. Intracellular Glassy State in Dry Seeds: A State for Survival

As previously stated, orthodox seeds can survive almost in anhydrobiosis. During seed maturation, the progressive loss of water induces the formation of a so-called “cytoplasmic glass”, an amorphous and viscous matrix resembling a solid-like state where thermobility and relaxation rates of molecules are severely slowed down (reviewed in [169]). A glass is a thermodynamically unstable solid state with an extremely high viscosity. Its formation is promoted by a low cell water content and is associated with improved storage stability [170,171]. A glassy matrix consists of soluble sugars, which immobilize macromolecules offering protection to membranes and proteins [170,172]. Thereby, with anhydrobiosis the cytoplasmic viscosity increases, the diffusion of water and O_2_ is suppressed, and the rates of all possible chemical reactions are dramatically reduced. In other words, cells of desiccation-tolerant organisms undergo the transition from the “liquid state” to the “glass state”, having the “rubbery state” as an intermediary. Thus, molecular mobility is extremely reduced, metabolic activity ceases, and only spontaneous chemical reactions causing cellular deterioration, such as direct oxidative attack (i.e., not enzyme catalyzed), molecular crowding, and the resulting Maillard reactions, appear to be possible causes of molecular damage [173,174]. In summary, the “glass state” is necessary to prevent cellular oxidative damage and maintain the native structure of macromolecules and membranes. Accordingly, membranes are considered a primary target of desiccation injury and its stabilization is a key mechanism for desiccation tolerance [175].

The triggering of desiccation causes: an increase in the concentration of intracellular solutes and alterations in its proximity and mobility; coalescence of oil bodies; loss of membrane integrity; and protein misfolding [87]. As stated in the appropriate section of this review (see Section 4), these cell abnormalities are prevented in desiccation-tolerant tissues by the presence of special molecules such as chaperones (i.e., LEAs and sHSPs) and oleosines [91,127,133,176]. sHSPs are known to play a role in the glassy-matrix formation, function as molecular chaperones that facilitate protein folding, and prevent irreversible protein aggregation. Plants synthesize at least 21 different types of sHSPs. On the other hand, lipid bodies protect plant lipid reserves against oxidation and hydrolysis until seed germination and seedling establishment. They can be stabilized by specific structural proteins, namely, the oleosins and caleosins, which act as natural solvents.

Those cellular components dependent on water to maintain their structure/function are protected during anhydrobiosis using so-called “water replacement” by specific, non-reducing oligosaccharides [177,178]. That is, the replacement of water by certain sugars becomes important upon further drying. It is thought that, in combination with highly hydrophilic proteins (i.e., LEAs), these non-reducing oligosaccharides can also enhance the quality and persistence of the “glass state” due to the capacity of oligosaccharides to form glasses [179,180,181]. That is, while the level of non-reducing sugars is low in fully hydrated cells, in dehydrated cells it is high. In summary, immobilization of the cytoplasm in a stable multicomponent glassy-matrix is of utmost importance. In other words, the cytoplasmic phase transitions from liquid-to-viscous-to-glass are thought to increasingly impede deleterious biochemical reactions while progressively dampening respiration [180,182].

To overcome the stress caused by strong removal of cellular water, in addition to accumulating compatible solutes (i.e., soluble sugars), cells of desiccation-tolerant pteridophytes and Angiosperms increase vacuolation and activate CW folding [41]. As described in Section 3.2, CW folding is essential for structural preservation of tissues and is species-specific. On seed drying, there is a reduction in glucose and an increase in galactose substitutions to the xyloglucans and it has been proposed that cleavage, or partial cleavage, of the long-chained xyloglucan units into shorter, more flexible ones allows for wall folding. The soluble sugars replace water in the membranes and macromolecules, fill and stabilize vacuoles, and generate a vitrificated cytoplasm [183]. The vitrification can be conceptualized as the transformation of hydrophilic cytoplasmic molecules in a “glass state” during desiccation [184]. Vitrification is a general property of sugars. This biological glass (i.e., liquid with an extremely high viscosity) immobilizes macromolecules thereby inhibiting most reactions, especially those that are catalyzed by enzymes, and prevents denaturation or other structural disruptions [27,161,185]. The “glass state” confers stability preserving the structural and functional integrity of macromolecules (e.g., enzymes). Thus, the secondary structure of proteins in dry viable seeds of several species appears to be very stable and remains preserved after several decades of storage [169,186]. Intracellular glasses exhibit a high molecular packing and slow mobility, resembling glasses made of mixtures of proteins and sugars. However, as early as the 1980s, the possibility that the cytoplasm of drying seeds could enter a “glass state” as a defense mechanism was analyzed and discussed [187,188]. Sucrose, the principal protective sugar, trehalose, raffinose-family oligosaccharides such as raffinose, stachyose and verbascose, and infrequent sugars as octulose, are involved in desiccation tolerance [46,88]. Increasing intracellular trehalose (nonreducing disaccharide) is sufficient to confer desiccation tolerance to *Saccharomyces cerevisiae*. That is, unlike plants, trehalose will be sufficient in conferring desiccation tolerance in the absence of other stress effectors.

Fundamental metabolic aspects such as transcription and translation can occur in water levels below 30% [41,43,44]. This remarkable fact has been demonstrated with great clarity in the desiccation-tolerant lichen *Flavoparmelia caperata* [189]. In this species, enzyme activity occurs at 0.17 g H^2^O/g DW, ceasing between 0.12 g and 0.08 g H^2^O/g, whilst the cytoplasm acquires an amorphous “rubbery” state at 0.17 g H^2^O/g DW due to its viscosity being five times higher than in the liquid state. The cytoplasm achieves an amorphous “glass state” at 0.03 g H^2^O/g DW, at which point no metabolic activity is recorded. On the other hand, at around 0.1 g H_2_O/g DW, the cytoplasm of *P. sativum* drying at 20 °C seeds vitrifies and enters the “glass state”. Dry seed longevity can be related to this feature [180]. As previously indicated, seeds accumulate both non-reducing sugars and LEA proteins during maturation. The interaction of sugars and LEA was demonstrated in wheat [190]. Using a proteomic approach, a subset of LEA proteins has been identified in relation to survival in the dry state, making them possible candidates for the stabilization of the “glass state” [191]. There is experimental evidence showing that LEA proteins from different groups acquire α-helix after complete drying [192]. These findings suggest that these proteins form α-helix in the dry seed.

## 7. ABI3 Is a Master Regulator of Desiccation Tolerance in Seeds

The acquisition of desiccation tolerance comprises highly coordinated molecular events such as the suppression of photosynthesis [93] and energy metabolism, and accumulation of protectants (i.e., LEA proteins, antioxidants, and soluble sugars). These events are tightly regulated by hormones (e.g., ABA) and TFs (e.g., ABI3) [34]. Interestingly, genes responsible for synthesis and signaling of ABA are found in basal land plants (i.e., bryophytes) and were likely important in the acquisition of desiccation tolerance during plant evolution [193,194,195]. ABI3 and ABI5 represent the most important players, involved in the control of seed maturation, metabolism of raffinose family oligosaccharides, and expression of *LEA* genes [35]. Specifically, ABI3 has the capacity of activating a cascade of diverse developmental and metabolic genes for seed maturation. *LEA* gene expression in seeds is tightly associated with ABA signaling given that promoters of approximately 82% of all *LEA* genes in *Arabidopsis* contain the ABA-responsive element (ABRE) motif [33,150]. *ABI3* expression is repressed in seeds initiating germination. In *Arabidopsis* seeds, chlorophyll degradation is under the control of ABI3 [196]. It is interesting to note that Arabidopsis *ABI3* and *LEC1* LOF mutants produce seeds that lose their viability during desiccation or during the first few weeks after harvest [29]. In other words, ABI3-regulated LEA protein abundance linked to desiccation tolerance in *M. truncatula* [29]. However, knowledge of how the genetic alterations that cause the various phenotypes take place during seed development and drying is still unknown.

ABI3 and ABI5 are two TFs that mediate desiccation tolerance in seeds through a highly conserved gene regulatory network [116,197,198]. ABI3 presents the highest number of correlations with desiccation-tolerance-related genes [101]. ABI3 and ABI5 showed opposing expression patterns during *C. australe* seed development compared with *M. truncatula*. Although ABI5 has no effect on seed dormancy and does not affect its level, it negatively regulates seed germination [199]. During desiccation, some resurrection species degrade chlorophyll and disassemble chloroplasts (poikilochlorophylly). The degradation of chlorophyll during the maturation (i.e., late stage) is common among orthodox seeds, perhaps to avoid the generation of ROS during subsequent long periods of storage in the dry state [60]. The disappearance of chlorophylls takes place before the viable orthodox seeds reach the dry state. Chlorophyll breakdown is impaired in the seed maturation mutants, particularly the severe alleles of *abi3* in which chlorophyll degradation does not take place and mature seed possesses green cotyledons [29,196]. These *ABI3* seed maturation mutants failed to acquire desiccation tolerance and were barely storable [130]. Recent results indicate that the gene regulatory network organized by ABI3 in the seeds and leaves of resurrection plants are evolutionarily conserved [44]. ABI3 regulates expression of *FUS3* in the embryonic axis and cotyledons and indirectly participates in the accumulation of sHSPs by positive regulation of HSFA9 TF. Interestingly, induction of seed dormancy and desiccation tolerance in *Arabidopsis thaliana* is indirectly correlated with ABI3 by regulation of sHSP factor HSFA9. The ABI3 knockout lines lack detectable levels of HSFA9-mRNAs and proteins. In summary, ABI3 is a master regulator of LEA protective proteins (see Figure 1 from [200]). On the other hand, *MtABI3*, *MtABI4*, *MtABI5,* and *MtAP2/EREBP* genes from the model legume *Medicago truncatula* were found to be highly connected with desiccation-tolerant genes and are therefore good candidates as desiccation-tolerance regulators [83,85,197]. MtABI3 is one of the TFs most connected to desiccation tolerance. So much so, ABI3 is the only TF involved in the acquisition of desiccation tolerance during early seed maturation and the re-induction of desiccation tolerance in germinated seeds. Interestingly, a large proportion of genes related to *MtABI3* in the network were identified as direct targets of ABI3 in *Arabidopsis thaliana* [197]. Likewise, *MtABI5* was found to be a hub in the gene network regulating seed survival in the seed dry state [85]. Several mutants in ABA signaling were shown to be compromised in their ability to re-induce desiccation tolerance in germinated seeds. Some observations have shown that the acquisition of desiccation tolerance during the seed maturation period is different from the re-induction of desiccation tolerance in germinated seeds [201]. Together, ABA is a questionless hormone involved in induction of desiccation tolerance and it was suggested that ABA signaling via the ABI3 pathway is highly conserved in the establishment of desiccation tolerance [83,201]. Finally, during seed desiccation, the size of the nuclei markedly reduces, and the chromatin undergoes a high but reversible condensation (Figure 3 from [202]). This condensation is probably not induced by desiccation per se but by a regulated phenomenon controlled by ABI3 [202,203]. In Arabidopsis, ABI3 has been implicated in activation of *HSP17.4* during seed development. This member of the sHSP family is not detected in the *abi3-6* knockout mutant. However, notable levels of *HSP17.4* were present in *lec1-2* and *fus3-3* mutants [133]. For further clarification, see Section 1.2 from [129]. Interestingly, ectopic expression of LEC2, FUS3, or ABI3 in the single- or double-mutant backgrounds of the other two regulators was unable to initiate desiccation tolerance, suggesting that all three regulators are required to activate the expression of TFs directly involved in desiccation tolerance [53]. The fact that expression of desiccation-related genes mediated by both ABA and ABI3 are conserved between *Physcomitrella patens* and Arabidopsis (i.e., from bryophytes to Angiosperm seeds) suggests a highly conserved gene regulatory network for protecting cells from desiccation among land plants.

## 8. Concluding Remarks and Perspectives for the Future

During desiccation, sensitive species start to die below an approximate relative water content of 55%, whereas tolerant species do not. Cells of all orthodox seeds must possess a core set of mechanisms to protect them from desiccation- and rehydration-induced damage. The importance of tolerance to desiccation is not only limited to basic research aimed at understanding the mechanisms that underlie this adaptive and strongly conserved feature at the evolutionary level. Additionally, the study of desiccation tolerance may generate agricultural (i.e., drought tolerance) applications with clear economic repercussions. Therefore, it is important to understand even more how fully orthodox seeds survive severe water deficits and apply this knowledge to generate more tolerant and robust crops through the application of emerging biotechnologies. Although in this century notable advances have been made in the knowledge of desiccation tolerance in orthodox seeds (see the present update), a good number of unknowns remain to be solved. One of the causes of the slowdown in the knowledge of this outstanding process is because the desiccation tolerance is triggered during seed maturation, a phase controlled by several hormonal signals within which ABA has a special impact. In addition, the ABA signaling network in the fully orthodox seeds still has serious gaps.

Most desiccation-sensitive plants possess tolerant seeds, which implies that the genomic information for desiccation tolerance is present in these species but is expressed only in their dispersion organs. Thus, transcriptomic studies are pivotal to complement the genomic data. As stated above, some protocols should be addressed, and several questions answered to show why a fully orthodox dry seed remains viable over time.

Thus, critical questions to address in the anhydrobiosis field include:

- Whether a single stress effector is sufficient to promote desiccation tolerance;
- Are the regulatory subnetworks that control vegetative desiccation tolerance in resurrection plants similar to those controlling desiccation tolerance in orthodox seeds?;
- Could a core desiccation tolerance regulatory network have been conserved throughout plant evolution?;
- Explore whether desiccation tolerance is orchestrated by regulatory networks in which at least a common core of TFs has been conserved during plant evolution and to determine how it has been rewired several times to be activated in orthodox seeds and vegetative tissues;
- Do the gene regulatory networks of vegetative desiccation tolerance in resurrection plants also involve ABI3?;
- Confirm in several species that deletion of *ABI3* resulted in loss of desiccation tolerance induced by ABA;
- Check that expression of orthodox seed-specific genes related to LEA are also activated in desiccating vegetative tissue of resurrection plants, suggesting a similarity of regulation mechanisms between the resurrection plants and fully orthodox seeds;
- How are the differences between fully orthodox and recalcitrant seeds generated to respond to dryness?;
- Since *PLATZ1*, *PLATZ2*, and *AGL67* genes are essential for the acquisition of desiccation tolerance in Arabidopsis seeds, are these three genes conserved in Angiosperms?;
- Does the folding process similarly occur in both fully orthodox seeds and desiccating resurrection plants?;
- How were fully orthodox seed-specific gene networks induced in desiccation-tolerant vegetative tissues?

In summary, a strong research work is still necessary to shed light on the process of desiccation tolerance in seeds; however, as indicated, such studies are worthwhile.
